# Update on glycerol-3-phosphate acyltransferases: the roles in the development of insulin resistance

**DOI:** 10.1038/s41387-018-0045-x

**Published:** 2018-05-25

**Authors:** Jing Yu, Kim Loh, Zhi-yuan Song, He-qin Yang, Yi Zhang, Shu Lin

**Affiliations:** 1 Quanzhou First Hospital, Fujian Medical University, Fuzhou, China; 2 Quanzhou People’s Hospital, Quanzhou Medical College, Quanzhou, China; 3 St. Vincent’s Institute of Medical Research, Fitzroy, VIC 3065 Australia; 4 Department of Cardiology, Southwest Hospital, Third Military Medical University (Army Medical University), Chongqing, China; 5 Illawarra Health and Medical Research Institute, Wollongong, 2522 Australia

## Abstract

Glycerol-3-phosphate acyltransferase (GPAT) is the rate-limiting enzyme in the de novo pathway of glycerolipid synthesis. It catalyzes the conversion of glycerol-3-phosphate and long-chain acyl-CoA to lysophosphatidic acid. In mammals, four isoforms of GPATs have been identified based on subcellular localization, substrate preferences, and NEM sensitivity, and they have been classified into two groups, one including GPAT1 and GPAT2, which are localized in the mitochondrial outer membrane, and the other including GPAT3 and GPAT4, which are localized in the endoplasmic reticulum membrane. GPATs play a pivotal role in the regulation of triglyceride and phospholipid synthesis. Through gain-of-function and loss-of-function experiments, it has been confirmed that GPATs play a critical role in the development of obesity, hepatic steatosis, and insulin resistance. In line with this, the role of GPATs in metabolism was supported by studies using a GPAT inhibitor, FSG67. Additionally, the functional characteristics of GPATs and the relation between three isoforms (GPAT1, 3, and 4) and insulin resistance has been described in this review.

## Introduction

**Fig. 1:**
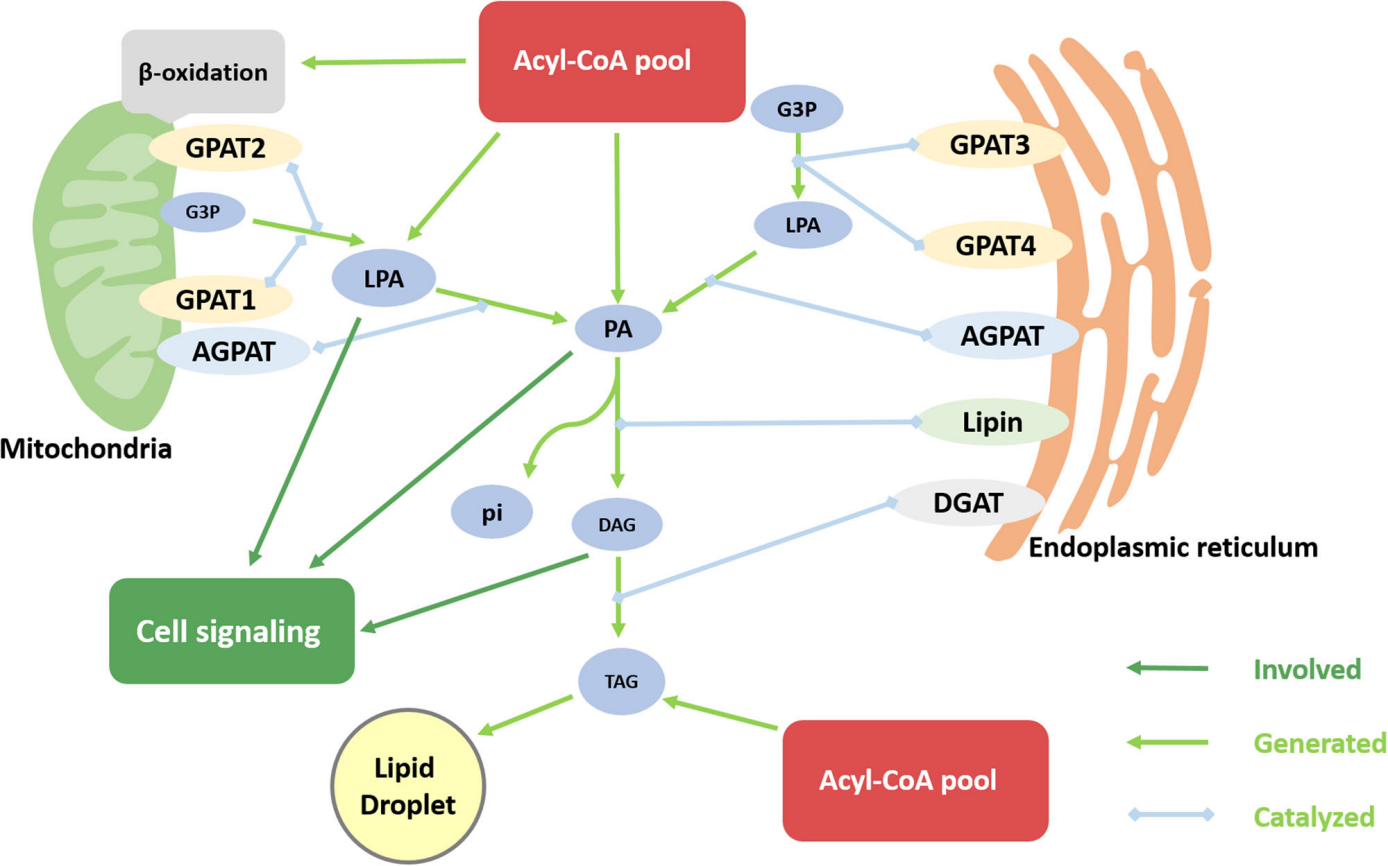
Glycerophospholipid pathway. Glycerol-3-phosphate acyltransferase 1 (GPAT1) and GPAT2 are localized in the mitochondrial outer membrane, while GPAT3, GPAT4, phosphatidic acid phosphatase (PAP/lipin), and diacylglycerol acyltransferase (DGAT) are localized in the endoplasmic reticulum (ER) membrane. In addition, 1-acyl glycerol-3-phosphate acyltransferase (AGPAT) is localized in both the mitochondrial outer membrane and the endoplasmic reticulum (ER) membrane. GPATs competitively catalyze acyl-CoA and glycerol-3-phosphate (G3P) to produce lysophosphatidic acid (LPA) and protect acyl-CoA from β-oxidation. Then, LPA and acyl-CoA are converted to phosphatidic acid (PA) by AGPAT. Consequently, PA is dephosphorylated by lipin to diacylglycerols (DAG). DAG and acyl-CoA are catalyzed by DGAT to form triacylglycerol (TAG). Furthermore, TAG synthesizes lipid droplets (LD). The intermediate products (LPA, PA, DAG) are responsible for intercellular signal transduction

Hypertriglyceridemia and lipid accumulation in non-adipose tissues are linked with the development of chronic metabolic diseases such as obesity, hepatic steatosis, insulin resistance, type 2 diabetes mellitus (T2DM), and cardiovascular diseases. Many studies have demonstrated the relation between insulin resistance and intra-cellular ectopic accumulation of triglycerides in non-adipose tissues^1–3^. GPATs catalyze the first step of synthesis of triacylglycerol (TAG), which also acts as the rate-limiting enzyme for the de novo pathway of glycerophospholipid synthesis due to the lowest specific activity^4^. In most tissues, TAG is produced through the glycerol phosphate pathway. However, in the small intestine, TAG synthesis occurs via the monoacylglycerol pathway, which contributes to the absorption of food-derived fat. There are a series of enzymes participating in the catalytic reaction of glycerol phosphate. GPATs catalyze the acylation of glycerol-3-phosphate (G3P) and acyl-CoA to synthesize lysophosphatidic acid (LPA). Subsequently, LPA will be converted into phosphatidic acid (PA), and this conversion is catalyzed by the 1-acyl glycerol-3-phosphate acyltransferase (AGPAT) family. Phosphatidic acid phosphatase (PAPase, also known as lipin) catalyzes dephosphorylation of PA to form diacylglycerols (DAG). The conversion of DAG to TAG is catalyzed by diacylglycerol acyltransferase (DGAT). Moreover, PA and DAG serve as the precursors of TAG in the biosynthesis pathway of glycerophospholipids, which participate in phospholipid remodeling^5^. Glycerophospholipids are the main components of biological membranes and play an important physiological role in animal cellular functions^3^ (Fig. 1). In mammals, four GPAT isoforms have been identified and can be further divided into two categories, mitochondrial and microsomal GPATs, which are localized in the mitochondrial outer membrane and endoplasmic reticulum (ER), respectively (Fig. 1)^6^.

T2DM is recognized as a serious metabolic disease and its incidence is increasing dramatically worldwide. It accounts for 90–95% of total diabetes cases and is often caused by insulin resistance coupled with a failure of the beta-cell to compensate due to impaired function, leading to hyperglycemia and subsequent glucose toxicity. In T2DM, insulin resistance is characterized by a decrease in the insulin responsiveness of the target tissues of insulin, particularly in liver, fat, and skeletal muscles. A reduction in insulin sensitivity in peripheral insulin-responsive target cells may contribute to impaired glucose homeostasis. In recent studies, the pathogenesis of insulin resistance and T2DM have been shown to be linked to lipid deposition in the liver^7^. In this review, we describe the link between several GPAT isoforms (such as GPAT1, 3, and 4) and insulin resistance.

## Mitochondrial GPAT1

### Structure and properties of GPAT1

Mitochondrial GPAT1 was the first isoform cloned into a mammalian vector system. Like other GPAT isoforms, GPAT1 possesses a conserved acyltransferase domain and two transmembrane domains, with the NH_2_– and COOH– terminal domains facing the cytosol, and forms a stem loop structure in the mitochondrial intermembrane space. The active site of acyltransferase was close to the NH_2_-terminal domain toward the cytosol, while the COOH-terminal domain is suggested to have a regulatory function^8^.

Mitochondrial GPAT1 activity, which has a huge impact on the regulation of TAG synthesis, is responsible for 30–50% of the total GPAT activity in the liver^9^ and approximately 30% of the total activity in the heart^10^. Mitochondrial GPAT1 is resistant to inactivation induced by sulfhydryl-group modifying reagents like N-ethylmaleimide (NEM). GPAT1 preferentially catalyzes saturated fatty acids (FAs) (palmitate acid or C16:0) and selectively transfers acyl-CoA to the sn-1 position of G3P. Subsequently, LPA, PA, and DAG are produced, as described previously, in the glycerophospholipid pathway. These intermediate substrates serve as the critical component of the ubiquitous biological membranes and mediate intercellular signal transduction.

### Regulation of GPAT1

GPAT1 is regulated at the transcriptional and post-transcriptional levels, and is highly expressed in adipose tissues and liver cells. It has been shown that carbohydrate response element-binding protein (ChREBP) and sterol regulatory element-binding protein-1c (SREBP-1c) are involved in the transcriptional upregulation of GPAT1 levels under increased dietary intake of carbohydrate and increased levels of insulin in plasma^11^. LXR (liver X receptor) when activated by its agonist could increase GPAT1 expression. During the differentiation of adipocytes, GPAT1 mRNA expression increases as a result of regulation of nuclear transcription factor-Y (NF-Y) and SREBP-1c^11, 12^. Insulin and adenosine 5′-monophosphate-activated protein kinase (AMPK) can also regulate the GPAT1 enzyme activity at the post-transcriptional level. Administration of insulin immediately increases the *K*_m_ and *V*_*m*ax_ of GPAT1 via protein phosphorylation. Conversely, AMPK, a key enzyme in the regulation of cellular energy homeostasis, phosphorylates GPAT1 to inhibit its enzyme activity^13^.

### Novel functional characteristics of GPAT1

Besides catalyzing glycerolipid synthesis, GPAT1 also critically regulates physiological functions in a number of specific cells types and tissues/organs such as adipose tissues and the liver^2^. GPAT1 plays a substantial role in modulating the cytokine production and proliferation of murine T-lymphocytes^14^. The T-cell phenotype under GPAT1 deficiency is comparable to that in an older mice, where GPAT1 activity decreases largely due to immune depression and results in increased susceptibility to infections^15^. A recent study has found that T-cells harvested from GPAT1^−/−^ mice showed significant reduction in interleukin-2 secretion upon antigen stimulation, and induced apoptosis accompanied by altered mass and composition of phospholipids^16^. LPA produced as a result of increased expression of GPAT1 via glucosamine prevents mouse embryonic stem cells from hypoxia-induced apoptosis through the mammalian target of repamycin (mTOR) activation^17^. Recent study reported that LPA also is a pivotal factor in mitochondrial fusion, which is produced as an intermediate substrate by mitochondrial GPAT1^18^. The infection with coxsackie-virus-B3 (CVB) in GPAT1^−/−^ mice sharply increased viral titers of CVB in both the liver and heart with high levels of proinflammatory cytokines^19^. This study also demonstrated the higher mortality due to cardiac pathology and the dysregulation of specific immune cells in the absence of GPAT1 with CVB infection^19^. A recent study found that the expression of mitochondrial GPAT, both at the mRNA and protein levels, was downregulated when targeted by upregulated miRNAs, such as miR-23b, in Epstein-Barr virus-infected B-cells under several culture conditions^20^. The alteration of lipid composition in GPAT1^−/−^ mice decreases the susceptibility to carcinogen-induced liver tumorigenesis^21^. Furthermore, although the changes in cellular metabolism associated with increased GPAT1 expression lead to overall ameliorative survival in breast cancer, the precise mechanism remains unclear^22^.

### GPAT1 and insulin resistance

Empirical evidence from animal models and in vitro experiments using rodent hepatocytes, either by overexpressing or knocking-out of GPAT1, has established the association of hepatic TAG accumulation with insulin resistance and T2DM. Previously, it has been shown that increased flux of lipid intermediates through GPAT1 overexpression causes hepatic insulin resistance without genetic or dietary-induced obesity (DIO)^23^. GPAT1 overexpression in rat hepatocytes reduced FA oxidation and competitively promoted de novo glycerolipid synthesis. Zhang et al. illustrated that overexpression of GPAT1 mediated by adenoviruses in rats increases levels of lipid intermediates (such as LPA, PA, and DAG), and subsequently activates the PKC-ε pathway and leads to impaired insulin signaling in the liver, which results in peripheral and hepatic insulin resistance^24^. In addition, GPAT1 deficiency in *ob/ob* mice led to a decrease in hepatic steatosis, TAG (~59%), and DAG (~74%), leading to the improvement of hepatic and systemic insulin sensitivity. The GPAT1-null mice also exhibited significant decreased plasma glucose levels and lowered the plasma TAG content in *ob/ob* background; however, the levels of acyl-CoA were observed to be elevated^25, 26^. Furthermore, recent studies have demonstrated that the overexpression of GPAT1 leads to impaired insulin signaling, reduced insulin-induced suppression of gluconeogenesis, substantially prevented mTOR complex2 (mTORC2) activity, and disassembled the link of mTOR/rictor mediated by PA, which induced peripheral and hepatic insulin resistance^24, 27^. In summary, mitochondrial GPAT1 is a critical regulator of TAG metabolism and systemic energy homeostasis; however, long-term high-fat-fed GPAT1^−/−^ mice did not show any beneficial metabolic phenotype^28^, indicating the complexity of the role of GPAT1 in metabolism under an over-nutrition state.

## Mitochondrial **GPAT2**

### Structure, properties, and regulation of GPAT2

Mitochondrial GPAT2 is the most mysterious isoform of the GPATs in mammals. GPAT2 was first detected in GPAT1^−/−^ mice^29^. GPAT2 is similar to GPAT1 in terms of structure and molecular mass. The two proteins located in the mitochondria share high homology. The NH_2_-terminal domain of GPAT2 associates with the active site, which possesses the maximum homology to that of GPAT1^8^. Compared to mitochondrial GPAT1, mitochondrial GPAT2 is sensitive to NEM. In the GPAT/AGPAT family, the acyltransferase contains four well-conserved domains (motif I–IV), which could catalyze the transferase reaction and are involved in binding to the substrate (acyl acceptor, e.g., G3P). The motif IV of GPAT2 is restricted to the mitochondrial membrane; however, the remaining acyltransferase motifs are exposed to the cytoplasmic side of the mitochondria or ER^30^.

GPAT2 is mainly expressed in pachytene spermatocytes of testis and may esterify G3P and LPA in the testis, whereas mtGPAT1 is primarily expressed in lipogenic tissues^31^. GPAT2 is also expressed in several types of human cancer-derived cells and its role in tumorigenesis is well characterized^32^. By contrast, the role of GPAT2 in lipid metabolism remains a matter of debate. Interestingly, the expression of GPAT2 is downregulated when 3T3-L1 cells differentiate into adipocytes, while GPAT1, 3, and 4 are upregulated (10-fold, >60, and 5-fold, respectively)^33^. A previous study showed that GPAT2 expression was unchanged in fasted or fasted-refed rodents, further implying that GPAT2 is unrelated to TAG synthesis or energy storage in the liver, and its transcription might not be under the regulation of SREBP1 or ChREBP^34^. Recent research indicated that retinoic acid activates GPAT2 expression in TM4 cells in vitro. The study shows that GPAT2 mRNA expression was increased and the content of TAG was significantly elevated in testis during sexual maturation in mice^33^, suggesting that GPAT2 may play an important role in the regulation of reproductive system.

### Novel functional characteristics of GPAT2

GPAT2 have a specific substrate preference for arachidonoyl-CoA^31^. A study on CHO-K1 cells reported that the expression and activity of GPAT2 are positively correlated with the TAG content incorporated by arachidonoyl acid in spermatogonia^31^. In rat and mouse testes, GPAT2 mRNA was detected only in primary spermatocytes, while GPAT2 protein was found in the meiotic and differentiated stages of spermatogenesis. GPAT2 regulates spermatogenesis and fertilization, including acrosome packaging, chromosome pairing, and specific gene expression^32^. It has been demonstrated that GPAT2 is closely linked to the cell cycle, DNA integrity maintenance, and epigenetic regulation^32^. GPAT2, also known as “cancer-testicular antigen”, is highly expressed in several cancer types (such as lung, melanoma, breast, and prostate cancer) and cancer-derived human cell lines, in which GPAT2 expression is associated with histological grading of the tumor. The expression level of GPAT2 promotes the proliferation, tumorigenicity, and migration rates of breast tumor cells^35^. GPAT2 is a murine MILI (mouse Piwi-like)-binding protein and is also involved in the primary biosynthesis of piRNA, which interacts with PIWI^36^. Many functions of GPAT2 may still be unexplored, such as an alternative enzyme activity and specific activity in testis.

## Microsomal GPAT3

### Structure and properties of GPAT3

Microsomal GPAT3/AGPAT10, which is localized in the ER, was originally named AGPAT8 due to its sequence similarity with AGPAT1 and 2, members of the AGPAT family. Subsequently, several studies revealed that it possesses GPAT activity instead of AGPAT activity, and this led to it being renamed as GPAT3^37^. Sukumaran et al.^38^ reported that GPAT3 exhibits AGPAT activity but lacks GPAT activity, and cloned it as AGPAT10. Overall, studies have shown that GPAT3 possesses both AGPAT and GPAT activities. Finally, it was renamed as GPAT3/AGPAT10.

Microsomal GPAT3 activity is sensitive to NEM and catalyzes a broad range of reactions using long-chain acyl-CoA as substrates, including saturated and unsaturated FAs. Sukumaran et al. also reported that GPAT3 uses oleoyl-CoA as the preferred substrate compared to all other acyl-CoAs such as palmitoyl-CoA, myristoyl-CoA, and stearoyl-CoA^38^. It was speculated that GPAT3 contains two transmembrane domains, and the active site of GPAT3 is located in the NH_2_-terminal domain^37^.

### Regulation of GPAT3

GPAT3 is predominantly expressed in the periepididymal white adipose tissue and small intestine of mice. In humans, GPAT3 is abundantly expressed in the kidney, heart, skeletal muscle, and thyroid gland^37^. In GPAT3^−/−^ mice, the total GPAT activity of the white adipose tissues decreased by 80%, while the overall liver GPAT activity remained unchanged. Another study by Muoio et al. demonstrated that AMPK activation in hepatocytes of rats leads to decreased mitochondrial GPAT activity but does not affect microsomal GPAT activity^39^. GPAT3 mRNA expression was significantly upregulated (about 60-fold) during 3T3-L1 adipocyte differentiation^37^. Above all, GPAT3 was recognized as a major isoform in the adipocytes for the synthesis of TAG^40^. GPAT3 activity increased in the white adipose tissue of diabetic mice after treatment with rosiglitazone, which is a PPAR-γ agonist^37^. GPAT3 and GPAT4 enzyme activity could also be regulated by insulin upon phosphorylation at the Thr and Ser residues^41^. On the contrary, knockdown of GPAT3 and 4 almost completely prevented the formation of lipid droplets (LD). It has been shown that deficiency of GPAT greatly reduces TAG synthesis and impairs adipogenesis^41^. The GPAT activity increased significantly after GPAT3 overexpression in COS-7 cells. By contrast, the activity of GPAT decreased dramatically with GPAT3-specific siRNA knockdown in 3T3-L1 cells, which directly inhibited lipid synthesis^37^. Collectively, it suggests that GPAT3 play a crucial role in lipid formation.

### Novel functional characteristics of GPAT3

A recent study has identified that GPAT3 served as a novel enzyme, playing critical roles in dietary lipid absorption, enteric and hepatic lipid homeostasis, as well as entero-endocrine hormone production^42^. Besides GPAT3 has been shown to be involved in the regulation of intestinal lipid metabolism, a recent study reported that thyroid-stimulating hormone (TSH) induces lipid production by activating the PPARγ/AMPK/GPAT3 pathway in a thyroxine-independent manner. Importantly, GPAT3 expression is also upregulated by TSH^43^. Moreover, during the stimulation of thermogenic and oxidative metabolism, the expression of GPAT1 and GPAT3 in inguinal adipocytes was increased by eicosapentaenoic acid to promote TAG synthesis with the inhibition of lipolysis^44^. Additionally, it was found that Seapolynol, which acts as an inhibitor of GPAT3, activated the AMPK pathway to inhibit lipid synthesis^45^. It is worth noting that SEIPIN (also known as BSCL2), a conserved regulator of the evolution of GPAT3 and 4, interacts with microsomal GPAT and subsequently downregulates its activity^46^.

### GPAT3 and insulin resistance

A recent study has revealed that GPAT3 plays a critical role in regulating glucose, energy, and lipid homeostasis^40^. GPAT3^−/−^ mice have increased energy expenditure, improved glucose homeostasis (lower fed glucose level, but not fasting glucose and insulin levels), decreased fat pad size, altered serum lipid levels (increased plasma free cholesterol, especially the low-density lipoprotein cholesterol level, and decreased plasma TAG), but enlarged liver size with increased plasma ALT/AST levels^40^. These data suggest that inhibition of GPAT3 may improve lipid and glucose metabolism, and provide beneficial effects in the treatment of metabolic diseases. Though increased energy expenditure was observed in both female and male mice; however, the study demonstrated a sexual dimorphism in the GPAT3-deficient phenotype. GPAT3^−/−^ female mice are protected from DIO and that the hepatic cholesterol metabolism is primarily altered only in GPAT3^−/−^ male mice^40^. These data confirmed the importance of GPAT3 in maintaining glucose and lipid homeostasis (Table 1).

**Table 1:** Summary of GPAT family enzymes

Gene	Other symbol	Molecular mass	Reaction to NEM	Subcellular localization	Tissue distribution		Ref.
GPAT1	/	94 kDa	Resistant	Mitochondria		BAT > WAT > liver > muscle > brain	^8^
GPAT2	/	88 kDa (human)89 kDa (mice)	Sensitive	Mitochondria		Testis > liver > adipose tissue	^29, 30^
GPAT3	AGPAT8/AGPAT10	50 kDa	Sensitive	Endoplasmic reticulum		Adipose tissue > small intestine > heart > brain > liver	^37, 38^
GPAT4	AGPAT6	52 kDa	Sensitive	Endoplasmic reticulum		BAT, testis > liver, kidney, brain, intestine, WAT	^47, 48^

*BAT* brown adipose tissue, *WAT* white adipose tissue

## Microsomal GPAT4

### Structure and properties of GPAT4

Microsomal GPAT4, originally named AGPAT6, was classified to be a member of the AGPAT family due to the high homology of its amino acid sequence with those of AGPAT1 and 2. However, it was found that AGPAT6 exhibited GPAT activity, but not AGPAT activity, and thus it was renamed as GPAT4^47^. With 66% amino acid identity with GPAT3, GPAT4 was identified as the second GPAT isoform, which was located in the ER membrane^48^. GPAT4, with a 456 amino acid-long sequence in humans, possesses a series of membrane insertion helices which form hairpin loops in the membrane or monolayer^49^. The active acyltransferase domain is located close to the C-terminal. There are approximately 100 amino acid domains in GPAT4 consisting of acyltransferase motifs I-IV, which are important for binding substrates and catalyzing the acyltransferase reactions^48^. The N and C termini of GPAT4 localized toward the cytoplasm are required for ER-to-LD relocalization^49^. It is speculated that GPAT4 has two or three multiple transmembrane helices and a 38 amino acid-long signal peptide^47^. GPAT4 overexpression in HEK293 cells increases NEM-sensitive GPAT activity. Microsomal GPAT4 is sensitive to NEM and can catalyze all kind of substrates^47^. Similar to GPAT1, GPAT4 has a mild preference for using C16: 0-CoA as a substrate compared to C18:0^24^. GPAT4 is highly expressed in the brown adipose tissue and testis in mice, with moderate expression in the liver and white adipose tissue. Histological analysis indicated that GPAT4 is also significantly expressed in renal tubular cells, the cerebellum, and the hippocampus in mice^48^. Importantly, it has been demonstrated that GPAT4 is widely expressed in human tissues^47^.

### GPAT4 and lactation

During lactation, GPAT4 mRNA is highly expressed in mammary gland epithelium, but not in the surrounding adipocytes of breast tissue. The levels of TAG and DAG of milk decreases by 90% in GPAT4-null mice due to an extraordinary decline in the number and size of fat droplets in mammary epithelial acinars and ducts. Pups nursed by GPAT4^−/−^ mice would die within 48 h after birth unless wild-type (WT) mice replaced the GPAT4^−/−^ mice to feed the pups^50^. These data suggested that GPAT4 plays a critical role in TAG synthesis during development.

### Novel functional characteristics of GPAT4

GPAT4 is also widely expressed postnatally in spermatocytes and around spermatids in mice testis, which might make a significant impact on spermatogenesis during the mid-meiosis. Overexpression of GPAT4 experiments showed that LPA produced by GPAT4 could stimulate mitogenic activity. Meanwhile, LPA serves as a mitogen regulating various cellular processes, such as cell proliferation and cytoskeletal reorganization^51^. Although, the relationship between TAG synthesis and LD growth and formation is unclear, Wilfling and colleagues found that GPAT4 relocalizes from the ER membrane to LDs during LD formation^49^. The reporter gene analysis showed that GPAT4 served as a glucocorticoid-regulated gene, which might contain at least one genome-wide glucocorticoid receptor-binding region, and mediate the response from the corresponding hormone^52^.

### The phenotype of GPAT4 knock-out mice

In GPAT4-deficient mice fed with a normal chow diet, GPAT4 activity was significantly reduced in the liver, brown adipose tissue, and mammary gland (45, 65, and ~90%, respectively) as compared to the control group, but was normal in inguinal adipose tissue. These data demonstrated that GPAT4 plays a unique role in TAG synthesis and maintains systemic energy balance during lactation in this tissue^48, 50, 53^. Like GPAT1 contributes to the primary mitochondrial GPAT activity in liver, GPAT4 also accounts for the primary microsomal GPAT activity in liver^41^. The body weight was significantly lower (25%) in GPAT4^−/−^ mice fed with normal chow diet compared to that of the mice in the WT group, suggesting that GPAT4 is a positive regulator of body weight. Compared to WT group, the changes related to beneficial metabolic performances were observed in GPAT4^−/−^ mice, including reduced hepatic and plasma TAG content (45–50%), and decreased epididymal adipose tissue, though the cholesterol content and inguinal adipose were unaltered^48^. As mentioned above, GPAT4^−/−^ mice were considered to be a model of selective subdermal lipodystrophy^48^. The loss of body weight and beneficial phenotypes of GPAT4^−/−^ mice might be considered to be associated with reduced content of adipose tissue or more energy expenditure; however, the exact mechanism remains unclear. Recently, it has been reported that high fat diet-fed GPAT4-null mice exhibited increased thermogenic gene expression in brown adipose tissue and performed a dramatic hyper-metabolism^54^. These data suggest that GPAT4 could limit the excessive exogenous FA oxidation and avoid the harmful hypermetabolic state^54^.

### GPAT4 and insulin resistance

**Fig. 2:**
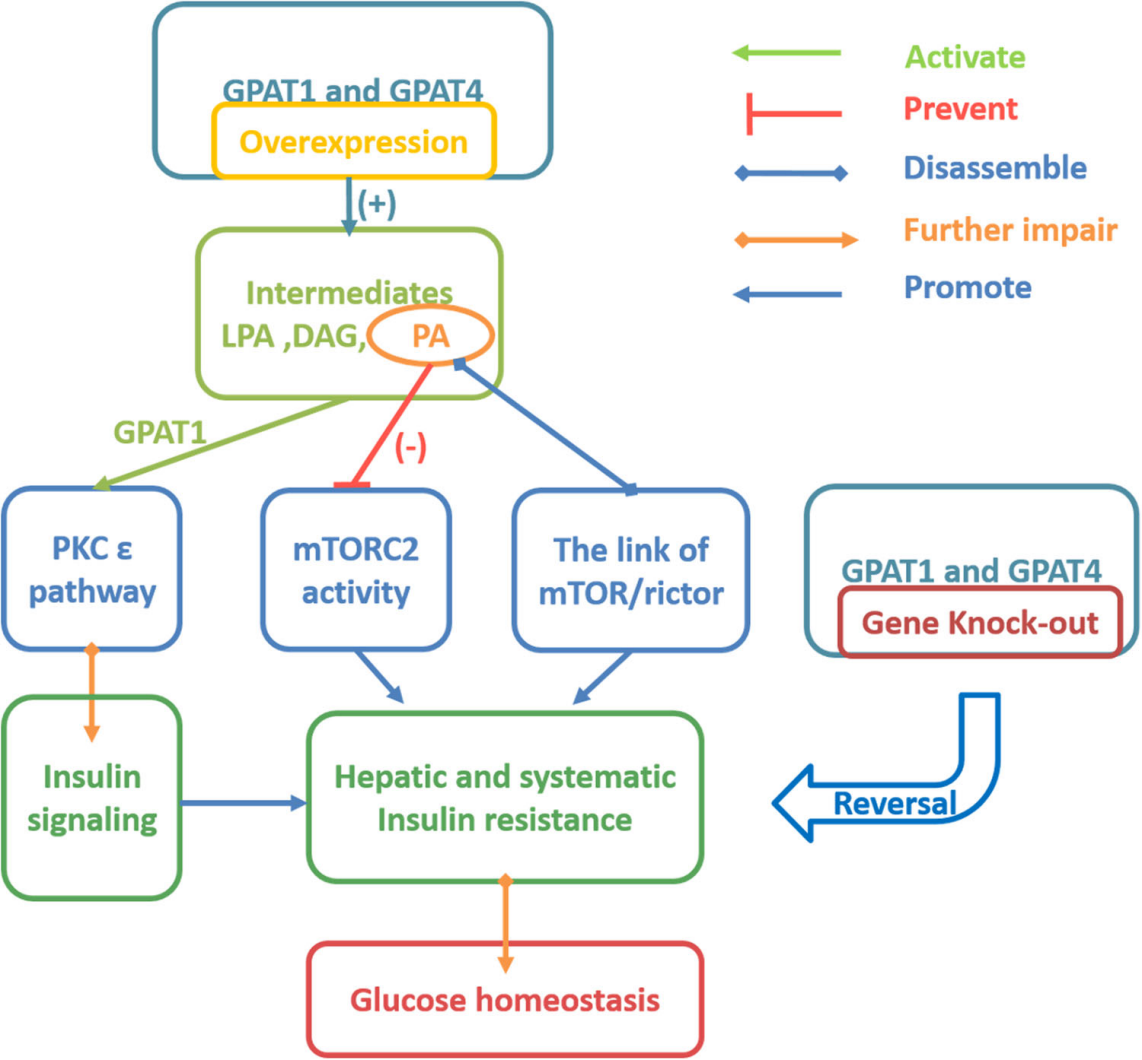
GPAT and insulin resistance. Glycerol-3-phosphate acyltransferase 1 and 4 (GPAT1 and GPAT4) overexpression impairs insulin signaling in mice. The increased intermediates by GPAT1 activated the PKCε pathway, and phosphatidic acid (PA) produced by overexpression of GPAT1 and GPAT4 interfere with the insulin signaling and inhibit the association between rictor and the mammalian target of rapamycin (mTOR) as well as mTORC2 (mTOR complex) activity in mice, which resulted in insulin resistance and impaired glucose homeostasis

Like mitochondrial GPAT1, GPAT4 is also associated with hepatic lipid accumulation and contributes to the development of insulin resistance. GPAT4 overexpression in hepatocytes led to impaired insulin-suppressed gluconeogenesis and decreased insulin-stimulated glycogen synthesis, as well as inhibited phosphorylation of Akt (Ser^473^ and Thr^308^) stimulated by insulin. Eventually, the changes led to the impaired glucose homeostasis^27^. In the same study, it was reported that overexpression of GPAT4 inhibited the association between rictor and the mTOR, and even the mTORC2 (mTOR complex 2) activity^27^. Like GPAT1, overexpression of GPAT4 increased the content of PA, which was produced in TAG synthesis pathway, particularly di16:0-PA. Compared with the control group (mice with C57BL/6J background which were littermates of GPAT4^−/−^ mice), the association of mTOR/rictor and mTORC2 activities in hepatocytes of GPAT4^−/−^ mice were increased, while the PA content declined dramatically; in particular, there was a sharp decrease in di16:0-PA. Overexpression of GPAT4 in HEK293 cells leads to increase the formation of LPA, PA, and DAG, while TAG levels were unchanged. Besides the increased immediate products, the report also illustrated that the lipid signal (such as di16:0PA) produced by GPAT4 interfered with the insulin signaling in hepatocytes of mice, which resulted in hepatic insulin resistance and impaired glucose homeostasis (Fig. 2)^27^. GPAT4-deficient mice had decreased body weight gain and subcutaneous fat pad, lowered hepatic and plasmatic TG levels, and improved insulin resistance compared to controls. In addition, GPAT4^−/−^ mice were protected from DIO and the development of insulin resistance in the liver and muscle cells through decreased content of di16:0 PA. These data suggest that GPAT4 might be a potential drug target for the prevention and treatment of obesity, insulin resistance, and type 2 diabetes^55^.

## Pharmacological inhibitor of GPATs

Small molecule inhibitors of GPAT, several compounds with series of benzoic and phosphonic acids, were designed and synthesized as potential pharmacological strategies for obesity and metabolic diseases linked with increased TAG synthesis by Wydysh and his colleagues^56, 57^. Besides in vivo and in vitro testing, it was found that these compounds, particularly 2-(nonylsulfonamido) benzoic acid or also known as FSG67, have a broad-spectrum inhibitory effect on GPAT activity^58^. Meanwhile, in pharmacological studies, it was found that FSG67 shows lower half maximal inhibitory concentration (IC_50_) to mitochondrial GPAT activity^56^.

With the administration of FSG67, the DIO mice exhibited a series of profitable phenotypes. Both DIO and lean mice had reduced their body weight and decreased the energy intake under the FSG67 treatment due to hypophagia, yet it does not appear to have conditional taste aversion^58^. During chronic low-dose treatment of FSG67, DIO mice showed reduced adiposity, increased FAβ-oxidation, and resistance to hypophagia-induced decline in metabolic rate, and even maintained the state of weight loss until the end of the study^58^. Kuhajda et al. also observed decreased plasma TAG levels, reduced fed plasma glucose, improved glucose tolerance, and increased insulin sensitivity in DIO mice treated with FSG67; however, there was no change in plasma cholesterol levels and fasting serum glucose levels. Meanwhile, small-sized white adipocytes and alleviated hepatic steatosis as well as lowered leptin levels were found in DIO mice chronically treated with FSG67. However, under the treatment of FSG67, DIO mice showed no subdermal lipodystrophy or abnormal appearance. Moreover, there is no evidence of deficient manifestation of sperm and spermatogonia as well. With the systemic administration of FSG67, the expression of lipogenic enzymes (such as GPAT, ACCI, and FAS) of lipogenic organs (like WAT, liver) was downregulated in DIO mice. Both acute and chronic treatment of FSG67 could alter the expression of hypothalamic neuropeptide in DIO mice, such as decreased Agouti-related peptide with high-dose FSG67 and declined neuropeptide Y with low-dose FSG67^58^. These data implied that the effect of FSG67 suppressed the hunger signals from central nervous system and produced series of beneficial metabolic phenotypes in high-fat-fed DIO mice.

FSG67, served as an anorexigenic compound, which enhanced the oxidative metabolism of FA (such as palmitic acid) by increasing CPT expression in primary hypothalamic neurons (PHN) cultured with excess lipid^59^. The data from cell culture research suggested that FSG67 did not downregulate the gene expression of the enzyme but directly inhibited GPAT activity. Moreover, it was speculated that FSG67 might promote FA oxidation through reducing esterification of acyl-CoA. The enhanced FA oxidation had increased reactive oxygen species (ROS) production in PHN. However, in this case, ROS production did not increase ER stress, initiate inflammation, or cause damage to mitochondrial health^59^. Moreover, the FSG67, inhibitor of GPAT suppresses the lipid synthesis in 3T3-L1^58^. Taken together, the data from cell and animal experiments imply that FSG67 treatment may prove to be beneficial to systemic metabolism (such as weight loss, reduced food intake, and increased FA oxidation).

## Conclusion

In this review, we describe and analyze the differences in functional characteristics, and tissue distribution of all GPAT isoforms, and the relation between several GPAT isoforms (GPAT1, 3, and 4) and insulin resistance. GPAT1 and 4 have an impact on glucose and lipid homeostasis in liver. GPAT2 plays a critical role in spermatogenesis and tumor development. GPAT3 is involved in TAG synthesis and is a major isoform of GPAT in adipose tissue. All GPAT isoforms are related to the synthesis and storage of TAG and participate in the synthesis of intracellular messengers. Overexpression and knockdown of GPAT in mice and cells have helped to demonstrate its function, including its importance in lipid metabolism and obesity-related diseases. The recently discovered functional characteristics of all GPATs have been described. In future, according to their distribution and features, we may evaluate a particular isoform of the enzyme as a therapeutic target for treatment of energy metabolism-related diseases. Moreover, treatment with GPAT inhibitor could prevent the development of adiposity and insulin resistance in DIO mice. This approach could be applied for the treatment of increased TAG-related diseases in humans. In addition, the transcription and regulation of GPAT genes involved in TAG synthesis still remains not fully understood. Further studies on GPAT function and regulation in individual tissues are required to fully establish GPAT as new drug target for the treatment of human metabolic diseases.
